# Supplementary material for: Netcombin: An algorithm for constructing optimal phylogenetic network from rooted triplets
Source: PLoS One. 2020 Sep 18;15(9):e0227842. doi: 10.1371/journal.pone.0227842 (PMC7500971; doi:10.1371/journal.pone.0227842)
Supplement: S4 File — (ZIP) [file pone.0227842.s004.zip › SequenceData-Triplet/sequenceData-10-20-30-0-Triplet/length 200, numseq 30, rate 10^-7/5 seq.pdf]

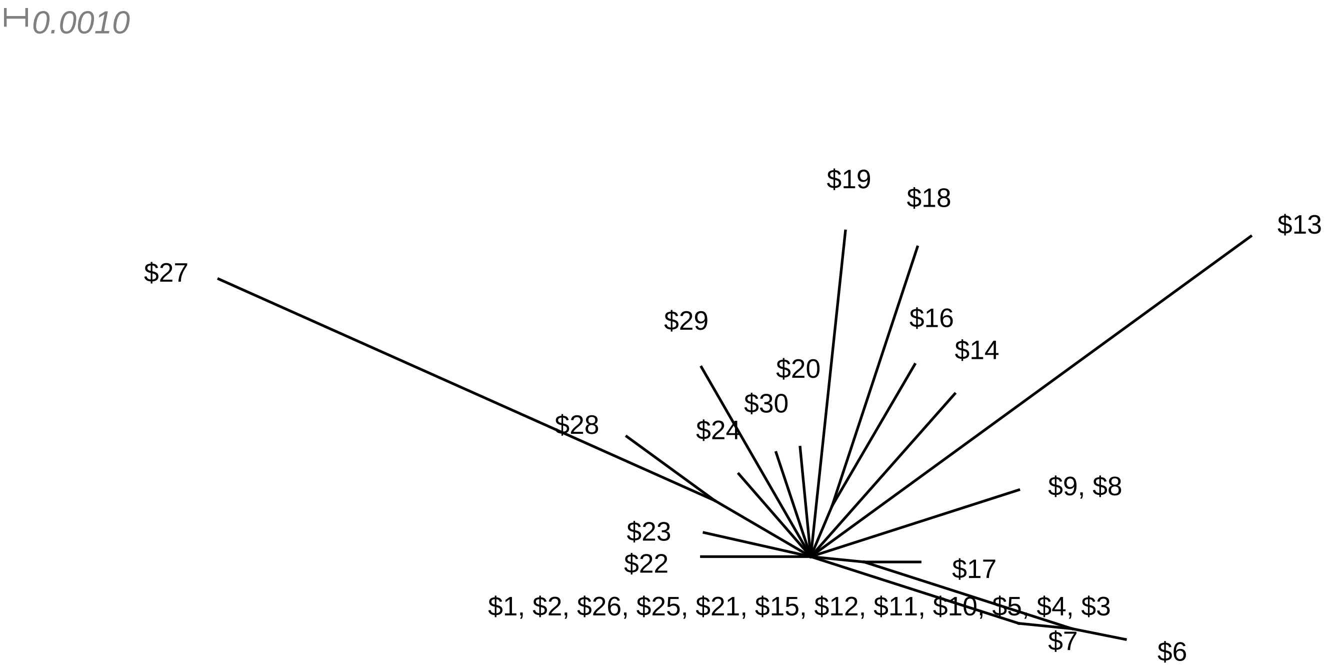
0.0010
$19
$18
$13
$27
$29
$16
$14
$20
$30
$28
$24
$9, $8
$23
$22
$17
$1, $2, $26, $25, $21, $15, $12, $11, $10, $5, $4, $3
$7
$6
